# The Chilean Maternal-Infant Cohort Study-II in the COVID-19 Era: A Study Protocol

**DOI:** 10.3389/fpubh.2022.904668

**Published:** 2022-07-14

**Authors:** María F. Mujica-Coopman, Camila Corvalán, Marcela Flores, María Luisa Garmendia

**Affiliations:** ^1^Public Nutrition Department, Institute of Nutrition and Food Technology, University of Chile, Santiago, Chile; ^2^Corporación de Salud Municipal de Puente Alto, Santiago, Chile

**Keywords:** pregnancy, nutrition, COVID-19, maternal health, infant health, infant growth, mental health, dietary intake

## Abstract

**Background:**

Pregnancy is a critical developmental window in which optimal maternal nutrition and health are key for pregnancy and infant development. The COVID-19 pandemic is considered as a “natural experiment” in which maternal and infant nutrition and health challenges were faced especially in developing countries. Therefore, understanding the health consequences for mothers and infants living in the COVID-19 era is key to revisit public health measures focused on maternal and infant health. The current work aims to describe the design, methods, and descriptive information at recruitment and preliminary findings of the Chilean Maternal & Infant Cohort Study II (CHiMINCs-II) cohort.

**Methods:**

The CHiMINCs-II is an ongoing cohort that is part of the Chilean Maternal and Infant Nutrition Observatory of the South-East area of Santiago, Chile. In total, 1954 pregnant women beneficiaries of the public health systems and their offspring were recruited before 15 weeks of gestation and are followed across pregnancy (<15, 26–28, and 35–37 weeks of gestation) and up to 2 years of age in their offspring. Two studies are currently nested within the CHiMINCs-II cohort: (1) Breast Cancer Risk Assessment in Mothers (BRECAM) study, and (2) the CHiMINCs-COVID study. The primary objective of BRECAM study is to test the association between maternal metabolic indicators (i.e., insulin, glucose, insulin growth factor 1, and hemoglobin A1c concentrations) at early pregnancy (i.e., <15 and 26–28 weeks of gestation) and breast density 3 months after the cessation of lactation. For this purpose, we collect maternal obstetric, lifestyle, dietary intake, anthropometric, and biochemical information. The aim of the CHiMINCs-COVID study is to assess maternal dietary intake and mental health problems derived from the COVID-19 pandemic and their association with maternal and infant's health and nutrition. Thus, we collected detailed information on dietary behaviors, mental health, and COVID-related information at each trimester, along with neonatal and infant nutritional information.

**Discussion:**

The findings of this study will provide novel and critical information to better understand maternal nutritional status, mental health, as well as infant growth and nutrition during the COVID-19 era.

**Clinical Trial Registration:**

BRECAM study registration number NCT03920098 and CHiMINCs-COVID study registration number NCT01916603.

## Introduction

Pregnancy is a critical period in which maternal nutrition, lifestyle and obstetric factors are key for adequate fetal growth (1). Also, all these factors are critical to ensure healthy mother and offspring development and prevent adverse health consequences such as reproductive-related cancers further in life (2–4). Thus, there is a need of advancing on interventions that can show effectiveness under real-life conditions (5, 6). In 2014, we conducted the Chilean Maternal & Infant Cohort study (CHiMINCs), a cluster randomized-controlled trial in which we showed that a low-intensity and high-coverage nutritional intervention delivered through public primary care clinics under standard operating conditions, reduced gestational weight gain, particularly among obese women (7). However, there is limited research on several other conditions such as cancer in identifying modifiable pregnancy risk factors as well as for defining more appropriate clinical management during pregnancy.

Breast cancer (BC) is the leading cause of cancer-related mortality in women worldwide (8) of which higher breast density (BD) (i.e., the percentage of dense tissue of an entire breast) is one of the most important risk factors (9, 10). Interestingly, pregnancy is a critical period for BD reduction, which may be underlined by hormone and pregnancy-related effects in the mammary gland (1, 11, 12). The relationship between reproductive factors, such as full-term pregnancy, and BD has been widely described (13, 14); however, there is a lack of evidence of the relationship between maternal hormonal and metabolic indicators such as insulin and glucose concentrations during pregnancy and BD.

In Chile, the COVID-19 pandemic began in March 2020 generating, as in most of the world, severe health, and economic disruptions. Thus, the COVID-19 pandemic and its associated mitigation measures have become an unexpected “natural experiment” during which the population has faced financial instability (15), mental health challenges (16), and higher food insecurity (17, 18). Pregnancy is a vulnerable period of life and a plasticity developmental window (19). In this sense, an inadequate maternal dietary intake of essential nutrients (i.e., micronutrients) (20, 21), as well as mental health-related challenges (i.e., stress, depression, mood disorders) (22, 23), may lead to adverse health outcomes in the mother and the offspring (24). Moreover, there is increasing realization of the connections between food and nutrition security and mental health (25). Early estimations about the consequences of the COVID-19 pandemic have indicated that the COVID-19 pandemic will significantly contribute to child wasting and mortality (26) and mental health problems (27). Also, we speculate that in countries with alarming rates of obesity and mental health problems such as Chile, food insecurity and inadequate dietary intake (e.g., the lack of consumption of nutrient-dense foods and the higher consumption of ultra-processed foods) may increase the risk of the double burden of malnutrition in our population (28) as well as further deteriorate mental health. Thus, we considered the ongoing CHiMINCs-II cohort as a unique and novel opportunity to explore how the pandemic has impacted nutrition and health of pregnant women in a Latin-American country and how these will relate to future maternal and infant health, including a particular focus on BC, one of the most prevalent cancers in women worldwide. The CHiMINCs-II cohort has the general goal to contribute evidence regarding nutritional and health factors related to maternal and infant development and the development of non-communicable diseases and obesity further in life. The primary intention of the present work is to describe how the CHiMINCs-II cohort is (1) exploring the relationship between maternal metabolic and hormonal changes during pregnancy and risk factors of non-communicable diseases such as breast cancer and (2) exploring the relationship between maternal dietary intake and mental health and maternal and offspring nutritional status living in the COVID-19 era.

## Methods and Analysis

The general aim of the CHiMINCs-II cohort is to contribute to the identification of modifiable pregnancy risk markers of future maternal and offspring nutrition and health conditions; given that the CHiMINCs-II cohort takes place under a real-life observatory the ultimate goal of the cohort is that these findings can be translated into concrete actions on the Chilean public health care system and else-where through the modification of clinical guidelines and health care practices. Currently, two ongoing studies are nested within CHiMINCs-II cohort: (1) The Breast Cancer Risk Assessment in Mothers (BRECAM) and (2) The CHiMINCs-COVID study.

The BRECAM study aims to contribute evidence regarding the association between metabolic and hormone disturbances during pregnancy and BD. Considering that BD is one of the most well-known and modifiable risk factors of BC (9), the main goal of the BRECAM study is to provide evidence on how to prevent BC risk through reducing BD. *The primary aim of the BRECAM study is to evaluate the association between an altered metabolic milieu [i.e., high levels of glucose, hemoglobin A1c (HbA1c), insulin, and insulin growth factor-1, IGF-1] measured in all trimesters of pregnancy, and BD (% absolute fibro-glandular volume, AFGV and % percentage of fibroglandular volume, FGV) measured by dual-energy X-ray absorptiometry (DXA) 3 months after the cessation of lactation in a cohort of pregnant women*. We expect that pregnant woman with an altered metabolic milieu (i.e., higher levels of glucose, HbA1c, insulin, and IGF-1) will have higher BD (defined as higher AFGV and higher %FGV) measured through DXA 3 months after cessation of lactation, compared to women without metabolic disturbances.

The CHiMINCs-COVID study aims to provide critical evidence regarding changes in food habits, food intake and mental health in pregnant women living in the COVID-19 era. Specifically, we sought to *(1) evaluate maternal food security (FIES scale), feeding behaviors and food intake during pregnancy, (2) describe maternal mental health symptoms during pregnancy (i.e., depression, anxiety, COVID-related stress, alcohol and drugs use), (3) describe the self-assessed adherence of COVID-19 related mitigation measures, (4) explore the association between maternal dietary intake and maternal mental health with maternal and offspring health outcomes (e.g., maternal nutritional status, glycemic control, prematurity, offspring anthropometry and body composition)in mother-offspring dyad*. The expected results of the CHiMINCs-COVID study are that pregnant women will change their dietary intake (i.e., a lower intake of nutrient-dense food) because of the COVID-19 pandemic. These dietary changes will be associated with poorer maternal mental health and the nutritional status of the offspring.

### Study Design

**Figure 1:**
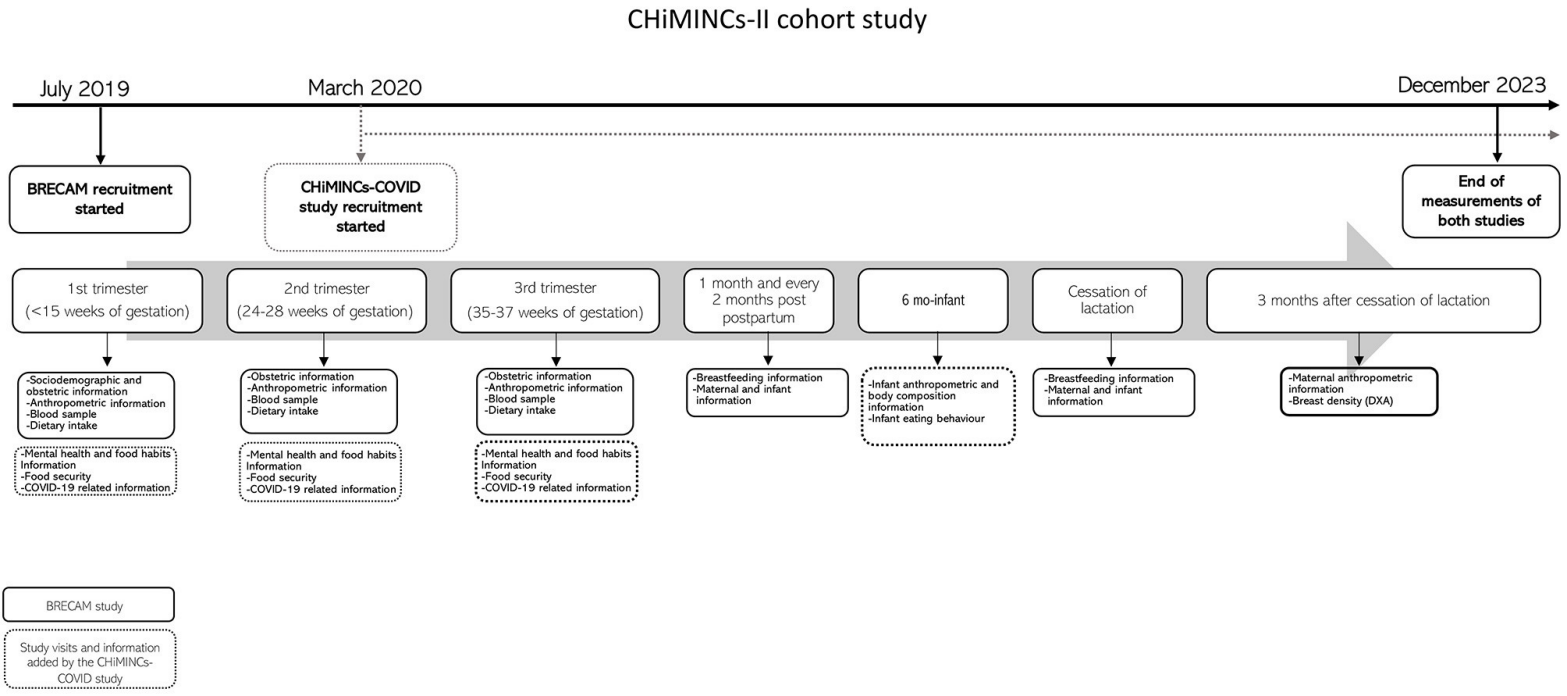
Timeline of the CHiMINCs-II cohort.

The CHiMINCs-II cohort is part of the Chilean Maternal and Child Nutrition Observatory (CHIMINO). Briefly, CHIMINO is a collaboration between the Institute of Nutrition and Food Technology (INTA), University of Chile, the Pontifical Catholic University of Chile, and the South East Metropolitan Health Service (SSMSO) that aims to provide evidence through interventional and observational studies for achieving healthy nutrition for mother and infants during the first 1,000 days, the early life window (29, 30). The BRECAM (NCT03920098) and CHiMINCs-COVID (NCT01916603) ongoing studies are nested within the CHiMINCs-II cohort, and both correspond to observational prospective cohort studies of pregnant women >18 y who seek prenatal care in any of the public health care centers (PHCC) of Puente Alto, the largest county in the South-East area of Santiago, Chile (Figure 1).

### Participants

Pregnant women receiving care in eight public primary health care units of Puente Alto County (largest county from the South-east area of Santiago, Chile) were invited to participate in the CHiMINCs-II cohort if they meet the following inclusion criteria: (1) >18 y, (2) <15 weeks of gestation at the first prenatal visit, (3) no intention to move outside of Santiago in the next 2 years. In the BRECAM study women were excluded if they had a high-risk pregnancy (i.e., preeclampsia, pre- existing diabetes) according to the National Guidelines (31), pre-existing cancer, family history of breast cancer while in the CHiMINCs-COVID study women were excluded, if they intended to migrate from the public to the private health care system (in Chile, about 20.6% pregnant women from the public system deliver their babies on private clinics) (32).

### Recruitment

The CHiMINCs-II cohort is an ongoing study, and we are currently collecting information and conducting research visits in pregnant women and their infants. Recruitment of the CHiMINCs-II cohort participants is part of a collaborative effort between the CHIMINO and the SSMSO. For the current cohort, pregnant women who received primary prenatal care within 8 PHCC located in Puente Alto County were approached between July 2019 and November 2021. From January 2022, we are approaching the mothers of the infants to ask permission for their infants to participate in a 6-mo study research visit. Information about potential participants of the study was obtained at the first prenatal appointment (<15 weeks of gestation) from the electronic clinical records of the PHCC. We also invited pregnant women living in the South-East area of Santiago to participate in the study *via* social media (i.e., Instagram, Facebook) using an online registration form. A field research assistant checked the eligibility of the potential participants according to the information provided on the online form, and then contacted the eligible participants at the PHCC or by phone to provide information of the studies. If they agreed to participate, research assistants scheduled a telephone research visit within the next 5 days. At the first appointment, inclusion and exclusion criteria were rechecked by trained dietitians; if the participant was confirmed as eligible for the cohort a recorded informed consent was obtained, first trimester measurements were collected and an online package with maternal and COVID-related questionnaires was sent to each participant.

### Follow-Up

Pregnant women were recruited <15 weeks of gestation and were followed across the second (24–28 weeks of gestation) and third trimester (35–37 weeks of gestation), delivery, 6-months post-partum, and 3 months after the cessation of lactation; telephone follow-up of milk-feeding behavior were also conducted every 2 months after birth (Figure 1).

All evaluations were conducted by trained dietitians; pregnancy evaluations were conducted by phone every trimester while the 6-months and 3 months after the cessation of lactation visits were conducted at the outpatient clinic of INTA. During pregnancy and at delivery biospecimens were collected (see Biological Specimen Collection for details) and milk-feeding behavior was collected by phone by trained personnel. In addition to the information collected at each visit, we had access to medical records that include routinely collected maternal and offspring information from prenatal appointments and at delivery. A summary of all the information collected in the CHiMINCs-II cohort is presented in Table 1.

**Table 1:** Study variables of the CHiMINCs -II cohort.

	**Instrument or source**	**Pre-pregnancy**	**≤15 wks**	**24–28 wks**	**35–37 wks**	**Delivery/ at birth**	**Postnatal 6 months**	**3 months after the cessation of lactation**	**Status**
**Maternal variables**									
**Anthropometry and body composition**									
Weight	Clinical records	✓	✓	✓	✓	✓			Collecting
Height	Clinical records		✓						Available
Blood pressure	Clinical records		✓	✓	✓				Available
Body composition	Body composition analyser TANITA BC-418							✓	Collecting
Breast composition	DXA							✓	Collecting
**Sociodemographic information**									
Personal and family history	Questionnaire and clinical records		✓						Available
Medical history	Questionnaire and clinical records		✓						Available
Past/current obstetric history	Questionnaire		✓	✓	✓				Available
**COVID-19 information**									
COVID-related information	Co-SPACE survey		✓	✓	✓				Available
**Mental health**									
Postnatal depression	Edinburgh postnatal depression scale		✓	✓	✓				Collecting
COVID-related anxiety	10 items of the pandemic anxiety		✓	✓	✓				Available
Stress	Pregnancy risk assessment monitoring system		✓	✓	✓				Available
**Dietary assessment and food security**									
Dietary intake	Single 24-h recall		✓	✓	✓				Collecting
Supplement use	Questionnaire	✓	✓	✓	✓				Collecting
Food security	Food insecurity experience scale		✓	✓	✓				Available
**Biological Specimen**									
Maternal samples	Plasma and serum samples		✓	✓	✓	✓			Collecting
Newborn feces						✓			Collecting
Cord blood samples						✓			Collecting
**Laboratory assessment**									
Glucose concentration	Fasting plasma sample		✓	✓	✓				Ongoing
Insulin, HbAc1, IGF-1, total estradiol, testosterone, and progesterone (*)	Fasting serum sample		✓	✓	✓				Ongoing
Total B-12 concentrations	Fasting serum samples		✓	✓					Ongoing
**Newborn information**									
Birth weight	Clinical records					✓			Collecting
Birth length	Clinical records					✓			Collecting
Head circumference	Clinical records					✓			Collecting
Gestational weeks at delivery	Clinical records					✓			Collecting
APGAR score	Clinical records					✓			Collecting
Clinical and family history	Clinical records					✓			Collecting
**6-mo infant information**									
Infant weight	Electronic scale (SECA 334 model)						✓		Collecting
Infant length	Infantometer (SECA 417 model)						✓		Collecting
Head circumference	Circumference measuring tape (SECA 212)						✓		Collecting
Body composition	PEAPOD						✓		Collecting
Clinical and family history	Questionnaire and clinical records						✓		Collecting
Infant feeding behavior	Infant & Young Feeding Practices questionnaire of WHO						✓		Collecting
	Baby Eating Behavior Questionnaire						✓		Collecting

### Obstetric, Sociodemographic, and Lifestyle Information

Maternal age (years), obstetric history (parity, pre-pregnancy weight), medical history (diabetes, hypertension, medication use, depression and anxiety, medication use), family medical history (depression, hypertension, type 2 diabetes, cancer, cardiovascular diseases, gestational diabetes, breast cancer), sociodemographic (marital status, family constellation, occupation, monthly family income) and lifestyle background information (physical activity, alcohol and drug consumption) was collected at the first study visit.

### Maternal Anthropometric, Blood Pressure, and Body Composition Information

Information on pre-pregnancy weight (kg), maternal height (m), weight (kg), and blood pressure (mmHg) of each participant was obtained from electronic clinical records of the PHCC. All the measurements are routinely collected by trained dietitians of the PHCC at each prenatal appointment, except for maternal height. We used the pre-pregnancy weight and height to calculate the pre-pregnancy body mass index (BMI). Pre-pregnancy BMI was used to classify pregnant women as underweight, normal weight, overweight and obese using the World Health Organization criteria. In addition, we measured maternal body composition after 3 months of lactation cessation using a monitor of body composition (Body composition analyser TANITA BC-418).

### Dietary Intake Information

At the first (*n* ~ 500), second (*n* ~ 1,200) and third-trimester (*n* ~ 1,000) study visits, dietary intake was collected by a trained dietitian using a semi-automated 24 h-recall, based on the multi-pass method (33). The multiple pass technique is a structured interview that enquires about all foods and liquids ingested the previous day from 00:00 h to 23:59 using the methodology of the 5-Step Multiple-Pass Method. Briefly, the interview is divided into five different steps which increase the chances of remembering usually forgotten food/beverage items. To increase the validity of the information collected, our group has developed a software, SER24H that uses the Food Atlas of the National Consumption Survey; ICC > 0.8 for test-retest. One dietitian will be specifically in charge of analyzing dietary data in terms of consistency and validity during the data collection period. Diet Analyses: SER24H provides the information of energy, macronutrients, and sodium for each food/beverage item, according to the weight/volume consumed and based on the nutrient composition databases of the United States Department of Agriculture (USDA) (34), homologated to Chilean foods (nutrient info obtained from the Chilean Food Composition Table) (35) and the nutrition fact panels of packaged food (collected in the context of the Food Environment Monitoring Project INFORMAS) (36, 37).

### COVID-Related Information

Information regarding COVID-19-related social isolation, occupation, social support, and diagnosis of COVID-19 of the participant or family members was collected in all trimesters using an online longitudinal survey that was developed as part of the COVID-19: Supporting Parents, Adolescents and Children during Epidemics (Co-SPACE) study (38). Additionally, self-assessed adherence to COVID-19 preventive measures promoted by the National Health authorities was measured using the Co-SPACE survey and the survey tool and guidance: rapid, simple, flexible behavioral insights on COVID-19 (39). Self-assessed knowledge of COVID-19, self-perception of risk of COVID-19 contagion, self-preventive behavior, sources of information related to COVID-19 news, and trust in COVID-19 related information provided by mass media was measured by the survey tool and guidance: rapid, simple, flexible behavioral insights on COVID-19 (39).

### Maternal Mental Health

Post-natal depression was measured using the self-administered Edinburgh Postnatal Depression Scale (EPDS) (40), whereas COVID-19-related anxiety was measured using 10 items of the Pandemic Anxiety Scale developed in the Co-SPACE study (38) during pregnancy. We also measured the occurrence of stressful situations in all trimesters of pregnancy using the Pregnancy Risk Assessment Monitoring System (41). In addition, we collected information related to alcohol consumption and recreational drugs use and sleeping habits (38) during COVID-19 pandemic.

### Food Security and Feeding Behaviors in the COVID-19 Era

For the collection of food security information, we used a reliable, valuable, and internationally validated food insecurity experience scale (FIES) developed by the Food and Agriculture Organization of the United States (FAO). Briefly, this scale was developed using data collected from more than 140 countries, and it provides information about food insecurity by asking the participants directly about their experience of food security (42). Specifically, the FIES measures food insecurity at the household or individual level. Furthermore, in a sub-sample of 350 pregnant women a food frequency questionnaire was used to collect information of the number on meals/days (i.e., breakfast, lunch, dinner), daily routine information (i.e., whether the participant had breakfast), snacks consumption, cooking skills, monthly consumption of rice, pasta, meat, fish, pulses, bread, cheese, milk, sugar, fruits, vegetables, and ultra-processed foods. In addition, we collected prenatal supplement use information (e.g., folic acid) before and during pregnancy, as well as the use of the national complementary food programs, sources of food supply during the COVID-19 pandemic (e.g., public markets), and the use of front-of-package nutrition labels to decide which foods to buy before and after the COVID-19 pandemic.

### Offspring Information at Delivery and 6 Months of Age

Information regarding birth weight (g), length (cm), head circumference (cm), weeks of gestation, APGAR score and maternal weight (kg) at delivery was obtained from clinic records. A 6-mo infant research visit is being conducted at INTA. Infant weight (kg) and length (cm) are measured using an electronic scale (SECA 334 model) and infantometer (SECA 417 model), whereas head circumference (cm) is measured using a circumference measuring tape (SECA 212) by trained dietitians. Infant body composition is determined by air-displacement plethysmography (PEA POD, Cosmed, Rome, Italy). The PEA POD body composition measurements have been validated against a four-compartment (4-C) reference model in infants (43), and their reliability and accuracy have been well-established. As part of this research visit, we also collect information regarding infant feeding behavior using a questionnaire based on the Infant & Young Feeding Practices and Baby Eating Behavior questionnaires (44).

### Biological Specimen Collection

*Maternal fasting blood samples* were routinely collected as part of the prenatal appointments at PHCC using an ethylenediaminetetraacetic acid (EDTA) and serum vacutainers by a certified phlebotomist in all trimesters. Blood samples were processed within the next 3 h at the county's laboratory. Plasma and serum samples were aliquoted and stored at −80 for further analyses. Additionally, as part of the CHiMINCs-COVID study non*-fasting maternal and cord blood samples* were collected at delivery using EDTA, serum vacutainer, and heparin tubes (i.e., for whole blood) for maternal samples, and EDTA and heparin tubes for cord blood samples. All samples were collected by a certified phlebotomist of the delivery team. Maternal blood samples were collected within 1–48 h before delivery, whereas cord blood samples were collected at delivery. Maternal and cord blood samples were processed within 6 h after collection at the hospital's laboratory. Maternal plasma, serum, whole blood and buffy coat samples, and cord blood buffy coat, plasma and whole blood samples were aliquoted (1 mL) in Eppendorf tubes (1.5 mL) and stored at −80°C for future analysis. Furthermore, *newborn fecal samples* (300 mg) were collected at 48 h after delivery using Eppendorf tubes (2 ml) and wooden sterile sticks. All the fecal samples were collected after the initiation of feeding (breastfeeding or formula) and were immediately stored at −80°C for further microbiome analysis.

### Biochemical Analyses

Insulin, HbA1c, IGF-1, total estradiol, testosterone, sex hormone binding globulin (SHBG), and progesterone concentrations will be determined in a sub-sample of pregnant women (*n* = 400) at least at one trimester during pregnancy. HbA1c concentration were measured by non-porous ion-exchange High-Performance Liquid Chromatography assay, whereas insulin and sex-hormone concentrations were measured by radioimmunoassay (Diagnostic Systems Laboratories). IGF-1 concentration were measured by chemiluminescent immunometric assay (Immulite 2000, Diagnostic Products Corporation). Additionally, we had access to glucose concentration information routinely determined by the glucose oxidase method (Photometric Instrument 4010; Roche) as part of the prenatal appointments at each trimester of pregnancy. As part of the CHiMINCs-COVID study, we will also measure total B-12 concentration in maternal serum samples collected at early-pregnancy (i.e., <31 weeks of gestation). Total B-12 concentration will be determined by chemiluminescent microparticle intrinsic factor assay.

### DXA

Breast composition is measured using DXA from GE Lunar Prodigy Bone Densitometer (GE Healthcare) calibrated to measure AFGV, %FGV and breast volume (BV) (45) at 3 months after the cessation of lactation. DXA measurements were conducted by a single trained research assistant and all participants were dressed only in a loose hospital gown in the chest area. Breast scans were taken with the participant in a decubitus mediolateral position and the nipple in a lateral position. Breast scans were exported as low-energy and high-energy attenuation images and breast composition data was calculated based on a two-compartment model (adipose and fibro-glandular tissue) using a software developed at University of California San Francisco. A single trained reader (intra and inter-rater ICC > 0.9), following a standard protocol, delineated the total projected breast area manually and the software estimates %FGV, AFGV, and BV automatically. Our group had previously shown a DXA precision of 2.8% for the measurement of BD in 200 adult women (46).

### Sample Size and Statistical Analysis

All the lifestyle, demographic, obstetric, biochemical, and COVID-related collected information was entered manually into REDCap by dietitians of the study. Dietary intake was entered into SER24H software by trained dietitians. The sample size was calculated based on the primary objective of the BRECAM study that was the one hypothesized to have the smaller effect size. Based on previous data from our group in pregnant women, the mean ± sd of %FGV was 29 ± 15%. A sample size of 700 women 3 months after breastfeeding cessation is sufficient to detect a difference of 4% in the mean of %FGV between groups (considering 140 women with high glucose levels and 560 with low glucose levels, ratio 1:4, based on our previous data in this population) using a two-sided test with 80% of power and a 5% level of significance. In addition, a sample size of 700 women will allow us to detect a difference of 6% in the mean of %FGV between groups [ratio high (5.7% or more) vs. low HbA1c levels 1:5]. Considering previous data in rates of abortion, loss to follow-up, drop-out and other exclusions previously mentioned (i.e., pre-eclampsia, preterm birth, gestational diabetes mellitus under treatment), we have the goal to recruit 1,500 pregnant women at the beginning of the pregnancy to have at least 700 women 3 months after the cessation of breastfeeding. For stratified analysis (by maternal adiposity or steroid hormone concentrations if interaction terms are statistically significant) we estimated that a final sample size of 300 women per strata would allow us to detect a difference in %FGV of 6% between groups.

The primary outcomes of the BRECAM study are (1) AFGV and (2) %FGV. The main predictors of the study will be: (1) glucose concentration, (2) HbA1c, (3) insulin concentration, and (4) IGF-1 levels during pregnancy. We will also collect data about potential confounders: maternal pre-pregnancy BMI, gestational weight gain, estradiol, progesterone, testosterone and SHBG levels, and initiation and duration of exclusive and predominant breastfeeding. Linear and logistic regression models will be used to test the association between the outcome variables (i.e., AFGV and %FGV) and predictors (i.e., glucose concentration, HbA1c, insulin concentration and IGF-1 levels).

In the case of the CHiMINCs-COVID study, 1,500 pregnant women would allow us to have sufficient power (0.8) to explore the associations between maternal dietary intake or mental health and (1) maternal nutritional status, (2) maternal metabolic control, and (3) offspring health outcomes (e.g., birth weight, prematurity). The primary maternal outcomes of the CHiMINCs-COVID study are (1) maternal weight gain during pregnancy, (2) glucose concentration, (3) HbAc1, (4) insulin concentration and (5) fulfillment of the mitigation COVID-19 measures. The primary infant outcomes are: (1) newborn anthropometric measurements (i.e., birth weight and birth length), (2) prematurity, (3) infant adiposity, and (4) infant anthropometric measurements (i.e., infant weight and length). The main predictors are (1) maternal dietary intake, (2) maternal mental health (i.e., depression and anxiety), (3) changes in maternal dietary intake, and (4) changes in maternal mental health. In addition, we will also collect information regarding potential confounders: (1) maternal education level, (2) income level, (3) pre-pregnancy nutritional status, and (4) history of depression. Linear and logistic regression models will be used to test the association between outcome maternal and infant outcome variables and maternal and infant predictor variables, respectively.

## Preliminary Results and Discussion

Recruitment of CHiMINCs-II started in July 2019. To date, we have recruited 1,954 pregnant women, which represents 130% of our goal (*n* = 1,500 pregnant women); we exceeded the original target sample because we observed that compliance with the study visits was lower than what we had originally estimated based on previous experience prior to the COVID pandemic. The collection rate of sociodemographic, medical, obstetric, anthropometric and blood pressure information was >80%. We have conducted 100% of the study visits at the first trimester, and we are currently following-up the recruited participants across the second and third trimester. At the time of writing this work, we have conducted 1,700 and 1,549 visits at the second and third trimester, respectively.

The recruitment for the CHiMINCs-COVID study started in March 2020. A total of 1,805 pregnant women were enrolled during COVID pandemic. We have collected sociodemographic, food intake, mental health, and COVID-19-related information in >84% of the participants at the first study visit (i.e., contacted by phone). Maternal characteristics of the CHiMINCs-II cohort at recruitment are presented in Table 2. The mean (±SD) age of the participants was 29 ± 6 years, most were single (53%) and had completed 12 y of education (71%). On average they were recruited at 12 weeks of gestation, and >70% of the participants had excess weight (BMI ≥ 25 kg/m^2^) before pregnancy.

**Table 2:** Baseline maternal characteristics of the CHiMINCs-II cohort participants (Chile, 2019–2022).

**CHiMINCS-II cohort**	***N* = 1,954^**a**^**
**Sociodemographic characteristics**
Age (y)	28.5 ± 5.98
Weeks of gestation	12.88 ± 4.45
**Civil status**, ***n*** **(%)**
Single	1,036 (53.02%)
Common law or married	886 (45.34%)
Divorced	17 (0.87%)
Widow	2 (0.10%)
**Education (y)**, ***n*** **(%)**
≤ 8 years	355 (18.17%)
8–12 years	1,038 (53.12%)
>12 years	556 (28.45%)
**Income ($USD)**, ***n*** **(%)**
≤ 410.00	521 (26.66%)
410.00–761.00	716 (36.64%)
≥ 761.00	502 (25.69%)
**Anthropometric characteristics**
Pre-gestational weight (kg)	73.40 ± 24.58
Height (cm)	159.01 ± 5.85
Pre-gestational body mass index	29.20 ± 6.16
**Nutritional status at recruitment**
Obesity	726 (38.97%)
Overweight	638 (34.25%)
Normal weight	481 (25.82%)
Underweight	18 (0.97%)
**Obstetric and medical history information**
Use of prenatal micronutrient supplementation, *n* (%)	362 (23.93%)
History of diabetes, *n* (%)	23 (1.18%)
History of hypertension, *n* (%)	47 (2.41%)
History of depressive symptoms (self-reported), *n* (%)	584 (29.89%)

a *Continous variables are expressed as mean ± standard deviation (SD). Categorical variables are expressed as frequency (%)*.

The CHiMINCs-II cohort has collected detailed information on maternal nutrition, mental health, and offspring health in mother-infant dyads experiencing the COVID-19 pandemic in Chile, which may represent the most challenging nutritional situation in the last decades. Considering that the CHiMINCs-II cohort is an ongoing we expect to collect a complete database of maternal and infant variables in 2,000 pregnant women and 600 infants (6 months of age), respectively.

Given the wealth of information we will be able to properly explore how nutrition and mental health impacts maternal and infant health, with a particular focus on BC-related outcomes. Furthermore, the availability of biological specimens (i.e., maternal blood samples, cord blood samples, and fecal samples) at different time points of the study offers the opportunity to explore critical research questions related to mechanisms underlying these relationships as well as identifying potential modifiable markers of disease. Ultimately, we expect that the CHiMINCs-II results will contribute to the update of clinical protocols and pregnancy risk-assessments to ensure better maternal and infant nutrition and health in the recovery of the pandemic.

## Ethics Statement

The CHiMINCs-II cohort nested studies were reviewed and approved by the Institutional Review Boards of INTA, University of Chile, and SSMSO. Participants provided informed consent before conducting any evaluation and understood that they could withdraw from the study at any moment. Given the restrictions because of the COVID-19 pandemic situation, we obtained written, electronic, or verbal consent (recorded by phone). All the consent forms were approved by the Ethics Committee at the Institute of Nutrition and Food Technology (INTA), University of Chile and SSMSO. Participants also provided informed consent to provide access to their clinical records.

## Author Contributions

MFM-C wrote the first draft of the manuscript. MG led the design and methods of the BRECAM study. CC led the design and methods of the CHiMINCs-COVID study. MF contributed to the coordination and data for the study. MG and CC were all responsible for the initial methodology described in the paper. All authors contributed to the manuscript writing and read and approved the final manuscript.

## Funding

The BRECAM study was funded by the national grant FONDECYT#1190532, and the CHiMINCs-COVID study was funded by the national grant ANID-COVID#0591 and FONDECYT#3210464. The funding sources had no role in the design of this study and will not have any role during its execution, analyses, interpretation of the data, or decision to submit results.

## Conflict of Interest

The authors declare that the research was conducted in the absence of any commercial or financial relationships that could be construed as a potential conflict of interest.

## Publisher's Note

All claims expressed in this article are solely those of the authors and do not necessarily represent those of their affiliated organizations, or those of the publisher, the editors and the reviewers. Any product that may be evaluated in this article, or claim that may be made by its manufacturer, is not guaranteed or endorsed by the publisher.

## Abbreviations

CHiMINCs: Chilean Maternal & Infant Cohort Study II
BRECAM: Breast Cancer Risk Assessment in Mothers
IGF-1: Insulin growth factor-1
BC: Breast cancer
BD: Breast density
HbA1c: Hemoglobin A1c
AFGV: % absolute fibro-glandular volume
FGV: % percentage of fibroglandular volume
DXA: dual-energy X-ray absorptiometry
CHIMINO: Chilean Maternal and Child Nutrition Observatory
INTA: Institute of Nutrition and Food Technology
SSMSO: Southeast Metropolitan Health Service
PHCC: public health care centers
BMI: body mass index
USDA: United States Department of Agriculture
EPDS: Edinburgh Postnatal Depression Scale
FAO: Food and Agriculture Organization of the United States
EDTA: ethylenediaminetetraacetic acid
SD: standard deviation.
